# Novel *LRP6* Mutations Causing Non-Syndromic Oligodontia

**DOI:** 10.3390/jpm12091401

**Published:** 2022-08-29

**Authors:** Yejin Lee, Wonseon Chae, Youn Jung Kim, Jung-Wook Kim

**Affiliations:** 1 Department of Pediatric Dentistry & DRI, School of Dentistry, Seoul National University, Seoul 03080, Korea; 2 Department of Molecular Genetics & DRI, School of Dentistry, Seoul National University, Seoul 03080, Korea

**Keywords:** hereditary, splicing mutation, oligodontia, *LRP6*, spontaneous mutation, genotype-phenotype relationship

## Abstract

The process of tooth formation is a series of reciprocal interactions between the ectoderm and mesoderm, and it is believed that many genetic factors are involved in this complex process. More than a dozen genes have been identified in non-syndromic tooth agenesis; however, the genetic etiology underlying tooth agenesis is not fully understood yet. In this study, we identified two novel *LRP6* mutations in two non-syndromic oligodontia families. Both probands had 16 and 17 missing teeth in their permanent dentition. Mutational analysis identified a de novo frameshift mutation by a 1-bp insertion in exon 9 (NM_002336.2: c.1870dupA, p.(Met624Asnfs*29)) and a splicing donor site mutation in intron 8 (c.1762+2T>C). An in vitro splicing assay confirmed the deletion of exon 8, and the deletion would result in a frameshift. Due to the premature termination codons introduced by the frameshift, both mutant transcripts would be degraded by nonsense-mediated mRNA decay, resulting in haploinsufficiency.

## 1. Introduction

Tooth formation is a result of a series of reciprocal ectodermal mesenchymal interactions [1]. Genetic defects or environmental assaults can cause tooth agenesis (TA) or a malformed tooth, such as peg lateralis, cone-shaped tooth, or taurodontism [2]. Due to the shared developmental origin, failure of tooth formation sometimes accompanies alterations in other ectodermally derived structures, such as the nails, hair, and sweat glands [3]. Therefore, TA can be classified as syndromic or non-syndromic TA, but the defects in the same gene can cause both TAs without a clear-cut boundary [4].

Oligodontia is a term that indicates a rare genetic condition in which six or more teeth (excluding wisdom teeth) are congenitally missing [5]. Anodontia, in which all teeth are missing, is extremely rare, but hypodontia, in which five or less teeth are missing, is relatively common. The pattern of TA, the locations of the conserved and missing teeth, could suggest candidate disease-causing genes [6]. Genetic heterogeneity and variable expressivity, however, make it hard to predict an exact culprit that causes selective tooth agenesis [7].

Even though the molecular pathology underlying TA is complex and is not fully understood yet, there are three major signaling pathways involved in TA: WNT/β-catenin, EDA/NF-κB, and TGF-β/BMP [8]. Aberrant signaling caused by mutant ligands, receptors, and any other components that relay the signal has been shown to cause TA, and the list includes *MSX1* (OMIM *142983, msh homeobox 1) [9], *PAX9* (OMIM *167416, paired box 9) [10], *AXIN2* (OMIM *604025, axin 2) [11], *EDA* (OMIM *300451, ectodysplasin A) [12], *EDAR* (OMIM *604095, ectodysplasin A receptor) [13], *EDARADD* (OMIM *606603, EDAR associated death domain) [14], *WNT10A* (OMIM *606268, Wnt family member 10A) [15], *WNT10B* (OMIM *601906, Wnt family member 10B) [16], *GREM2* (OMIM *608832, gremlin 2, DAN family BMP antagonist) [17], *BMP4* (OMIM *112262, bone morphogenetic protein 4) [18], *SMOC2* (OMIM *607223, SPARC-related modular calcium binding 2) [19], *LRP6* (OMIM *603507, LDL receptor-related protein 6) [20], and *KREMEN1* (OMIM *609898, kringle containing transmembrane protein 1) [21].

In this study, we recruited families with oligodontia and performed mutational analysis by whole-exome sequencing. The mutational analyses revealed two novel *LRP6* mutations, and an in vitro splicing assay verified the effect of the mutation. This report expands the mutational spectrum of the *LRP6* gene. A literature review with the identified mutations compared the effect of the *LRP6* mutations to advance our understanding of the molecular pathogenesis of TA.

## 2. Materials and Methods

### 2.1. Human Subject Enrollment

Protocols of the study and patient consent were reviewed and approved by the institutional review board of Seoul National University Dental Hospital (CRI05003G and 9 December 2021). Informed consent was obtained from all participating individuals after explaining the nature of the study. The pedigree was drawn with the family histories taken. Clinical examination and sample collection were performed according to the principles in the Declaration of Helsinki.

### 2.2. Genomic DNA Isolation and Whole-Exome Sequencing

Genomic DNA was isolated from 2 mL of peripheral blood by a conventional method with the NucleoSpin Blood L kit (Macherey-Nagel GmbH & Co., Düren, Germany) according to the manufacturer’s instructions. After measuring the quality and quantity using the NanoDrop1000 spectrophotometer (Thermo Fisher Scientific, Wilmington, DE, USA), the DNA samples of the probands were submitted for whole-exome sequencing (Yale Center for Mendelian Genomics, West Haven, CT, USA, and Theragen Etex Bio Institute, Suwon-si, Gyeonggi-do, Korea). After exome capturing, paired-end sequencing reads were generated.

### 2.3. Bioinformatic Analysis

Obtained sequencing reads (Table 1) were processed using a series of bioinformatic analyses as previously described [22]. Briefly, after trimming to remove the adapter sequences, the reads were aligned to the reference human genome assembly (hg38). Bioinformatics analysis programs, such as Samtools and Genome Analysis Tool Kit, were used to get a list of sequence variants [23,24]. The dbSNP build 147 database was used for the annotation of the sequence variants, and the annotated variants were filtered with a minor allele frequency of 0.01.

### 2.4. Sanger Sequencing

The identified mutations and the segregation among the family members were confirmed by Sanger sequencing. The following primer pairs for the *LRP6* gene were used: sense (5′-GTCCTTCTGTGCCCCTTTTA-3′) and antisense (5′-TCTCCCTTTTAGTCCCTAGCTTT-3′) primers for exon 9 and sense (5′-TCATCATGTAATTTTGAGAAAGCA-3′) and antisense (5′-TCTCCCTTTTAGTCCCTAGCTTT-3′) primers for exon 8. Sanger sequencing was performed for all participating family members at Macrogen (Seoul, Korea). The *LRP6* mutations were submitted to the ClinVar database (https://www.ncbi.nlm.nih.gov/clinvar/ (accessed on 26 July 2022), Accession ID: SCV002549922 and SCV002549923).

### 2.5. Human Identification and Paternity Test

DNA samples of the trio of family 1 (the proband and parents) were submitted for human identification (Macrogen). A short tandem repeat multiplex assay was performed with the AmpFISTR^®^Identifiler^®^ KIT (Applied Biosystems, Foster City, CA, USA) using 15 tetranucleotide repeat loci and the Amelogenin gender-determining marker: all thirteen of the required loci for the Combined DNA Index System and two additional loci (D2S1338 and D19S433). Genotyping data were obtained with the Applied Biosystems^®^ 3730/3730xl DNA Analyzer and analyzed with GeneMapper ID v3.2 (Applied Biosystems).

### 2.6. In Vitro Splicing Assay

The wild-type and mutant genomic fragments of the *LRP6* gene were amplified from a DNA sample of the proband of family 2 using the Pfu Plus 5x PCR Master mix (Elpis Biotech, Daejeon, Korea) and cloned into the TOPcloner Blunt V2 vector (Enzynomics, Seoul, Korea) using the same primers as in the exon 8 sequencing (including exons 8 and 9). After the sequences were confirmed, the wild-type and mutant fragments were subcloned into the pSPL3 splicing vector after double digestion with NotI and BamHI restriction endonucleases. COS-7 cells were transiently transfected with the wild-type and mutant pSPL3 vectors, and total RNA was isolated after 36 hours, and the cDNA was synthesized. RT-PCR (sense 5′-TCTGAGTCACCTGGACAACC-3′ and antisense 5′-AGGAAAGCCTCTGGGACAAT-3′) was performed with the EzPCR™ Basic 5x Master Mix (Elpis Biotech). Amplification bands were excised from an agarose gel following electrophoresis, purified, and characterized by direct DNA sequencing.

### 2.7. Dimensional In Silico Modeling

To better understand the molecular characteristics of the *LRP6* protein, three-dimensional modeling was performed with the PyMOL Molecular Graphics System (Version 1.8.2.3, Schrödinger, LLC., New York, NY, USA). Structure modules (3s94, 3s8z, 5gje, and 6l6r) were downloaded from the Research Collaboratory for Structural Bioinformatics (RCSB) Protein Data Bank (https://www.rcsb.org/ (accessed on 26 July 2022)).

## 3. Results

### 3.1. Clinical Phenotype and Mutational Analysis of Family 1

**Figure 1:**
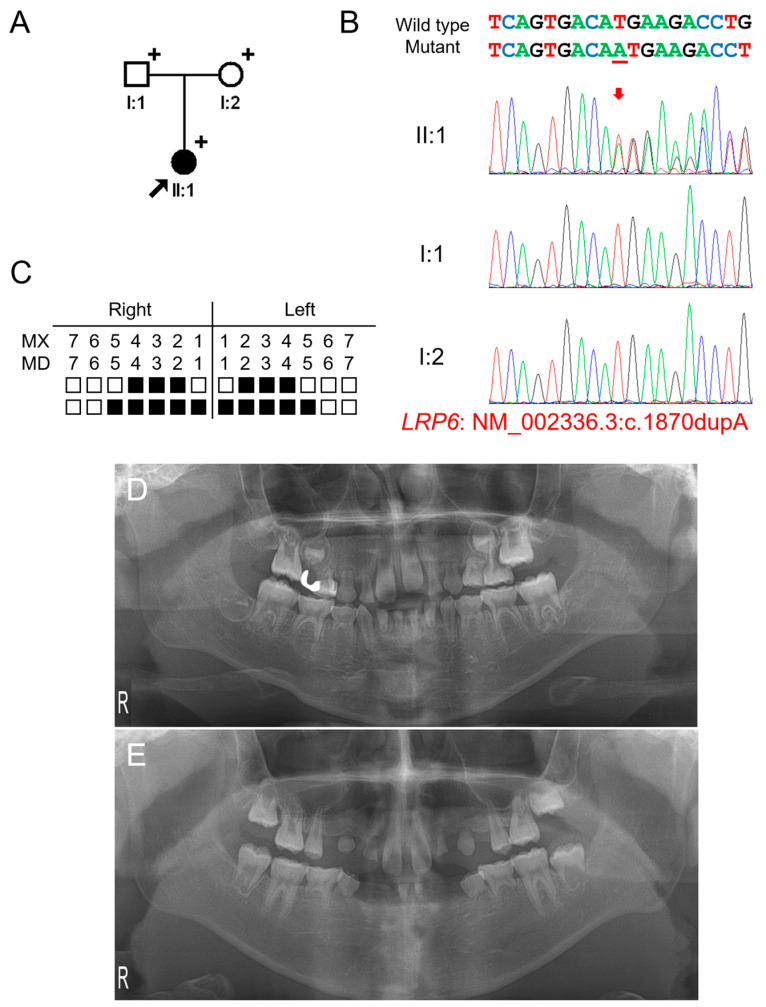
Pedigree, chromatograms, and panoramic radiographs of family 1. (**A**) Pedigree of family 1. Black symbols indicate affected individuals, and the proband is indicated by a black arrow. Plus signs above the symbols indicate participating individuals. (**B**) Sequencing chromatograms of the participating individuals of family 1. Wild-type and mutant nucleotide sequences are shown above the chromatograms. Nucleotide affected by the mutation is underlined. The location of the mutation is indicated with a red arrow. Individual identifications are indicated on the left side of each chromatogram. (**C**) Summary chart of the missing teeth of the proband. Black box indicates a missing tooth. Tooth number is shown above the boxes (MX: maxilla, MN: mandible). (**D**) Panoramic radiograph of the proband at age 5 years 11 months shows multiple missing teeth. Maxillary left first molar exhibits ectopic eruption. The maxillary first molars have taurodontism. (**E**) Panoramic radiograph of the proband at age 12 years 5 months.

The proband of family 1 was a 5-year-11-month-old female from a non-consanguineous Korean family (Figure 1). Pregnancy and delivery were uneventful, and she had no remarkable past medical history. Her deciduous dentition was normal without malformed or missing teeth; therefore, her family did not know about the missing multiple teeth in her permanent dentition. They were told about it when an examination was performed at a local dental clinic. The right maxillary second deciduous molar was exfoliated, and the distal-shoe space maintainer was placed. The left maxillary first molar exhibited an ectopic eruption with the resorption of the distal root of the left second deciduous molar. The maxillary first molars had taurodontism. She had 16 permanent teeth missing excluding the third molars (which were also missing), but no other syndromic features including oral exostosis. Her parents were normal without any missing teeth. Therefore, a recessive or de novo dominant mutation was suspected.

Mutational analysis of the whole-exome data resulted in three variants in genes related to TA: heterozygous WNT10A (NM_025216: c.637G>A, p.(Gly213Ser)), homozygous EDAR (NM_022336: c.1138A>C, p.(Ser380Arg)) and heterozygous *LRP6* (NM_002336.2: c.1870dupA, p.(Met624Asnfs*29)) variants. The WNT10A variant was inherited from the healthy mother and previously reported to cause autosomal recessive oligodontia [25]. Therefore, this heterozygous variant is not considered as a disease-causing mutation. The parents were heterozygous for the EDAR variant, and the proband was homozygous. This variant was previously reported as a disease-causing mutation to cause autosomal dominant inheritance with high conservation and in silico prediction values. However, it has been shown this variant exhibits an increased EDAR signaling output to a similar level of the variant p.(Val370Ala). Therefore, this variant also is not considered as a disease-causing mutation, even though it is homogeneous. The heterozygous *LRP6* variant was a 1-bp insertion in exon 9 of 23 exons and predicted to cause a change in the reading frame (p.(Met624Asnfs*29)). This frameshift would result in a premature termination codon (PTC) in the early exon; therefore, it would be degraded by the nonsense-mediated mRNA decay system (NMDS) [26], resulting in the haploinsufficiency of *LRP6*. This mutation was not found in the parents, and the paternity test confirmed that this mutation occurred spontaneously. Therefore, this frameshift mutation caused by a de novo 1-bp nucleotide insertion is believed to cause severe oligodontia in the proband.

### 3.2. Clinical Phenotype and Mutational Analysis of Family 2

**Figure 2:**
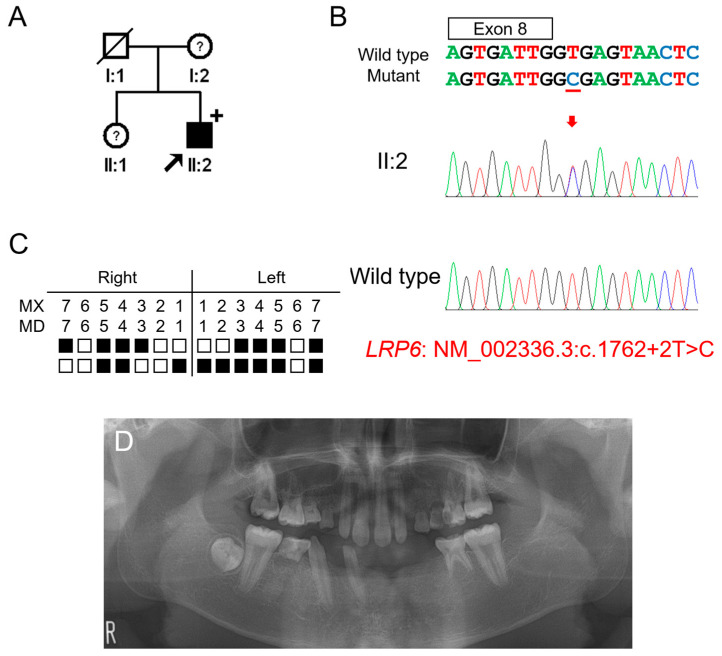
Pedigree, chromatograms and panoramic radiographs of family 2. (**A**) Pedigree of family 2. Black symbols indicate affected individuals, and the proband is indicated by a black arrow. Plus sign above the symbol indicates participating individuals. Symbols with a question mark inside indicate individuals whose phenotypes are not confirmed. (**B**) Sequencing chromatograms of the proband of family 2. Wild-type and mutant nucleotide sequences are shown above the chromatograms. Exon is indicated by a box above the nucleotide sequences. Nucleotide affected by the mutation is underlined. The location of the mutation is indicated with a red arrow. Individual identifications are indicated on the left side of each chromatogram. (**C**) Summary chart of the missing teeth of the proband. Black box indicates a missing tooth. Tooth number is shown above the boxes (MX: maxilla, MN: mandible). (**D**) Panoramic radiograph of the proband at age 14 years 5 months shows multiple missing teeth. First molars exhibit taurodontism.

The proband of family 2 was a 14-year-5-month-old male from a non-consanguineous Korean family (Figure 2). He had no other remarkable past medical history. His mother and sister were reported to have missing teeth; however, examination and confirmation were not available. He had 17 missing permanent teeth excluding his missing third molars. Maxillary lateral incisors were peg lateralis bilaterally, and the first molars showed the characteristic feature of taurodontism. Whether the deciduous teeth were missing could not be confirmed. There were no other syndromic features including oral exostosis.

**Figure 3:**
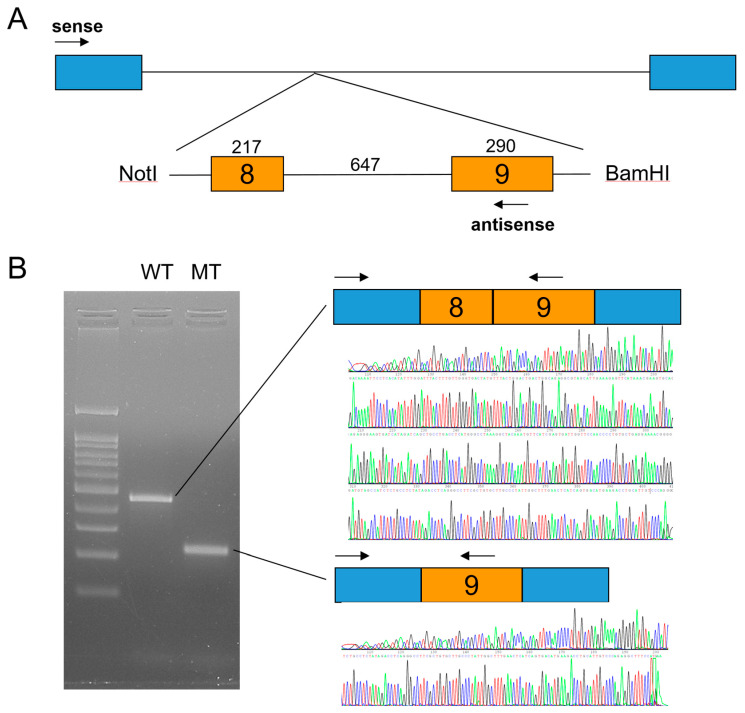
In vitro splicing assay. (**A**) Minigene cloning strategy. A genomic fragment including exons 8 and 9 of *LRP6* was subcloned into the pSPL3 vector with double digestion using NotI and BamHI restriction endonucleases. Boxes indicate exons, and horizontal lines indicate introns. The number of the exon is in the box, and the length of the exon and intron is shown above the boxes and line. Locations of the primer binding site are indicated with arrows (sense and antisense). (**B**) Agarose gel image of the splicing assay of the wild type (WT) and mutant (MT). Left lane is the DNA ladder. Wild-type and mutant names are shown above the gel image. Wild-type vector resulted in a normal splicing product including exons 8 and 9. Mutation (c.1762+2T>C) resulted in an exon 8 deletion. Sequencing chromatograms of the alternative splicing band are shown below.

Candidate gene sequencing of the MSX1, WNT10A, and EDA genes was performed but failed to identify any disease-causing mutations. Whole-exome sequencing revealed no other variants in the genes causing TA, except for an *LRP6* variant, a transitional change of thymine to cytosine at the canonical splicing donor site sequence (GT to GC) in intron 8 (NM_002336.2: c.1762+2T>C). An in vitro splicing assay confirmed the deletion of exon 8 during pre-mRNA splicing (Figure 3). Deletion of the 217-bp exon 8 would cause a change in the reading frame and introduce a PTC in exon 9. This mutant mRNA transcript would be degraded by the NMDS, resulting in the haploinsufficiency of *LRP6* as in the case of family 1.

## 4. Discussion

In this study, two novel *LRP6* mutations were identified, and the mutational effect was confirmed by an in vitro splicing assay. Both mutations would result in the haploinsufficiency caused by mutant mRNA degradation by the NMDS. Both probands exhibited severe TA (16 and 17 missing) in their permanent dentition. Usually, the TA phenotype caused by *LRP6* mutations does not, minimal if any (lateral incisors), exhibit any missing deciduous teeth [27]. Even though missing deciduous teeth could not be confirmed in the proband of family 2, the proband of family 1 does not have any missing deciduous teeth. A previous study reported taurodontism in about one-third of affected individuals [20], and the probands in our study also showed taurodontism: mandibular second molars in the proband of family 1 and all four first permanent molars in the proband of family 2. *LRP6* mutations related to tooth agenesis is listed in Table 2.

Expressivity is variable, especially wide in some missense mutations [33,38]; furthermore, an individual with incomplete penetrance was reported in a familial case with a frameshift mutation (c.1144_1145dupAG), which is supposed to be degraded by the NMDS [20]. A recent study demonstrated that there could be many more unknown genetic players involved in TA because mutations were identified in only about 25% of the study participants (12 out of 49 subjects) [36]. Additionally, a possible synergistic effect of the *LRP6* and *WNT10A* mutations has been also suggested [30]; therefore, it would be possible that multigenic effects among genes, even unknown and yet to be identified, are involved in TA.

Without proper functional studies, interpretation of an identified potential candidate variant sometimes misleads us to misunderstand the molecular pathogenesis of TA (including other complex disease entities), especially if the variant is rare and the amino acid encoded is highly conserved among species, because in silico predictions would favor the pathologic effect if so [42,43]. Due to the limitation of the repertoire of the known genes, it is possible that it might not be disease-causing but just very rare, and the disease is caused by other unknown gene(s).

**Figure 4:**
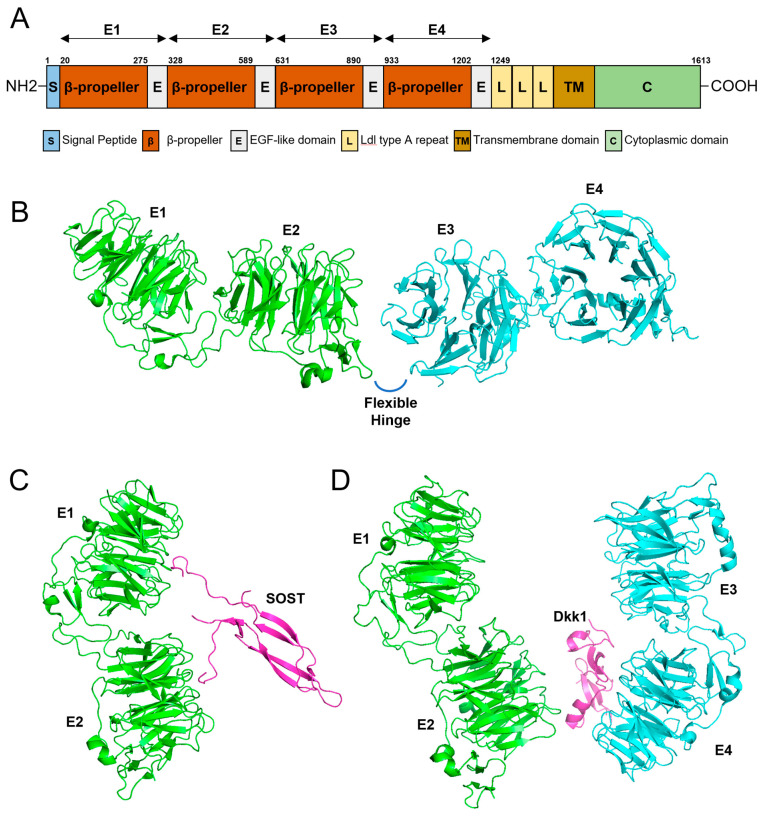
Gene diagram and 3D modeling. (**A**) Domain structure of *LRP6*. Amino acid numbers are shown above the diagram. Four β-propeller/EGF-like domain repeats (E1, E2, E3, and E4) are shown above the diagram with arrows. (**B**) 3D modeling of the four β-propeller/EGF-like domain repeats (E1, E2, E3, and E4). There is a flexible hinge between E2 and E3. (**C**) Three-dimensional modeling of E1 and E2 binding to SOST. (**D**) Three-dimensional modeling of the ectodomain of *LRP6* complexed with Dkk1.

The domain structure of the *LRP6* protein shows interesting features (Figure 4). *LRP6* is a single-pass transmembrane protein with an N-terminal ectodomain with four characteristic β-propeller/EGF-like domain repeats (E1, E2, E3, and E4) and a C-terminus cytoplasmic domain [20]. There is a hinge-like flexibility between the E1E2 and E3E4 structures, and this flexibility enables conformational freedom if there is no ligand bound [44]. This free movement hampers the unwanted formation of homo or heterobinding of *LRP6* to form an uncontrolled signaling platform [45]. With a ligand bound such as Wnt, restriction to free movement and conformational change enables the formation of oligomerization to convey controlled signals [46]. Interestingly, a conformational change caused by Dkk1 binding prevents oligomerization [44].

*LPR6* mutations sometimes accompany syndromic features such as sparse hair like in ectodermal dysplasia, a cleft lip and palate, hand preaxial polydactyly, and an orofacial cleft (OFC) (Table 2) [27,29,31,32,35,38]. The mutation in OFC is a deletion causing a frameshift in the last exon; however, due to the PTC location in the last exon, the mutant transcript would escape from the NMDS and produce a truncated protein with novel amino acids in the cytoplasmic C-terminal region [27]. This would be more harmful than the other frameshift mutations degraded by the NMDS by a dominant negative effect. Ligand binding would be normal with the intact ectodomain, but intracellular signaling would be disturbed or trigger a malfunction. Some mutations show interesting features, such as a high bone mass (with or without torus palatinus) with minimal TA (maxillary only or all four lateral incisors missing) [31,32]. The location of the mutations is in the E1 domain and may suggest involvement of certain factors to cause the high bone mass. Likewise, expanding the mutational spectrum of *LRP6* and related phenotypes may provide insight into the complex regulation and function of *LRP6*-mediated signaling pathways.

## 5. Conclusions

In this study, we recruited families with non-syndromic oligodontia and identified two novel *LRP6* mutations: a de novo frameshift mutation by a 1-bp insertion in exon 9 (c.1870dupA, p.(Met624Asnfs*29)) and a splicing donor site mutation in intron 8 (c.1762+2T>C). A minigene splicing assay confirmed that the effect of the splicing mutation and the mRNAs of both mutations are predicted to be degraded by the NMDS due to PTC. Further studies including genetic and functional studies are warranted to understand and characterize the complex mechanism of tooth formation.

## Figures and Tables

**Table 1:** Statistics for exome sequencing.

Sample	Total Reads	Mapping Rate (%)	Median Target Coverage	Coverage of Target Region (%)	Fraction of Target Covered with at Least	
					20X	10X
Family 1 II:1	82454353	99.9	80	96.3	92.6	95.0
Family 2 II:2	70852136	99.8	50	91.2	78.6	85.4

**Table 2:** *LRP6* mutations related to tooth agenesis.

Location	cDNA Change	Amino Acid Change	Domain	References	Additional Features
Exon 2	c.56C>T	p.(Ala19Val)	Signal peptide	[20]	
Exon 2	c.94C>T	p.(Arg32*)		[28]	
Exon 2	c.195dup	p.(Tyr66Ilefs*4)		[29]	
Exon 2	c.246A>T	p.(Lys82Asn)	E1-bP	[28]	
Exon 3	c.503T>G	p.(Met168Arg)	E1-bP	[30]	
Exon 3	c.517C>G	p.(Arg173Gly)	E1-bP	[27]	
Exon 3	c.553A>C	p.(Asn185His)	E1-bP	[31]	high bone mass, torus palatinus
Exon 3	c.602C>T	p.(Ala201Val)	E1-bP	[31]	high bone mass, torus palatinus
Exon 4	c.678-684delins	p.(His226_Phe228delinsGln)	E1-bP	[32]	high bone mass
Exon 4	c.711G>T	p.(Leu237Phe)	E1-bP	[33]	
Exon 6	c.1003C>T	p.(Arg335*)		[34]	
Exon 6	c.1095dup	p.(Asp366Argfs*13)		[29]	
Exon 6	c.1144_1145dup	p.(Ala383Glyfs*8)		[20]	
Exon 6	c.1154G>C	p.(Arg385Pro)	E2-bP	[35]	
Exon 6	c.1252T>C	p.(Tyr418His)	E2-bP	[27]	
Exon 7	c.1406C>T	p.(Pro469Leu)	E2-bP	[30]	
Exon 8	c.1603A>T	p.(Ile535Leu)	E2-bP	[36]	
Exon 8	c.1609G>A	p.(Gly537Arg)	E2-bP	[27]	
Exon 8	c.1681C>T	p.(Arg561*)		[29]	
Exon 8	c.1762+2T>C	p.?		This Report	
Exon 9	c.1779dup	p.(Glu594*)		[20]	
Exon 9	c.1870dup	p.(Met624Asnfs*29)		This Report	
Exon 9	c.1924dup	p.(p.Ile642Asnfs11*)		[37]	
Exon 10	c.2224_2225dup	p.(Leu742Phefs*7)		[20]	
Exon 10	c.2260G>C	p.(Ala754Pro)	E3-bP	[30]	
Exon 11	c.2292G>A	p.(Trp764*)		[29]	ectodermal dysplasia
Exon 12	c.2570G>A	p.(Arg857His)	E3-bP	[38]	sparse hair
Exon 12	c.2747G>T	p.(Cys916Phe)	E3-E	[34]	
Exon 13	c.2840T>C	p.(Met947Thr)	E4-bP	[35]	TA and Hand preaxial polydactyly
Intron 13	c.2994+1G>A	p.?		[27]	
Exon 14	c.3076C>T	p.(Arg1026Cys)	E4-bP	[36]	
Exon 15	c.3224A>G	p.(Asn1075Ser)	E4-bP	[30]	
Exon 15	c.3373C>T	p.(Arg1125*)		[39]	nsCLP
Exon 15	c.3388G>A	p.(Asp1130Asn)	E4-bP	[36]	
Intron 15	c.3398-2A>C	p.?		[27]	
Intron 16	c.3607+3_6del	p.?		[40]	
Exon 18	c.3754C>T	p.(Gln1252*)		[30]	
Intron 19	c.4082-2A>G	p.?		[27]	
Exon 23	c.4594del	p.(Cys1532Alafs*16)	ICD	[27]	OFC
Exons and introns 16–23	Interstitial loss of 290 kb in 12p13.2			[41]	

## Data Availability

The data presented in this study are openly available in ClinVar (http://www.ncbi.nlm.nih.gov/clinvar (accessed on 26 July 2022)), Accession ID: SCV002549922 and SCV002549923.
